# Hiding opinions from machine learning

**DOI:** 10.1093/pnasnexus/pgac256

**Published:** 2022-11-16

**Authors:** Marcin Waniek, Walid Magdy, Talal Rahwan

**Affiliations:** Computer Science, New York University Abu Dhabi, Abu Dhabi 129188, United Arab Emirates; School of Informatics, The University of Edinburgh, Edinburgh EH8 9YL, UK; Computer Science, New York University Abu Dhabi, Abu Dhabi 129188, United Arab Emirates

**Keywords:** Stance detection, privacy, machine learning, social media

## Abstract

Recent breakthroughs in machine learning and big data analysis are allowing our online activities to be scrutinized at an unprecedented scale, and our private information to be inferred without our consent or knowledge. Here, we focus on algorithms designed to infer the opinions of Twitter users toward a growing number of topics, and consider the possibility of modifying the profiles of these users in the hope of hiding their opinions from such algorithms. We ran a survey to understand the extent of this privacy threat, and found evidence suggesting that a significant proportion of Twitter users wish to avoid revealing at least some of their opinions about social, political, and religious issues. Moreover, our participants were unable to reliably identify the Twitter activities that reveal one’s opinion to such algorithms. Given these findings, we consider the possibility of *fighting AI with AI*, i.e., instead of relying on human intuition, people may have a better chance at hiding their opinion if they modify their Twitter profiles following advice from an automated assistant. We propose a heuristic that identifies which Twitter accounts the users should follow or mention in their tweets, and show that such a heuristic can effectively hide the user’s opinions. Altogether, our study highlights the risk associated with developing machine learning algorithms that analyze people’s profiles, and demonstrates the potential to develop countermeasures that preserve the basic right of choosing which of our opinions to share with the world.

Significance statement Our study suggests that a significant proportion of Twitter users wish to keep private their opinion about social, political, and religious issues. This is problematic in an age where machine learning algorithms are able to infer such opinions from the users’ profiles. Furthermore, we find that people cannot reliably identify the activities that reveal one’s opinion to such algorithms. These findings suggest the need for an automated assistant that would help Twitter users avoid exposing their opinions online. We evaluate possible ways of implementing such an assistant and find that introducing features indicating the opposite opinion can effectively mislead state-of-the-art machine learning algorithms. Our study represents a step toward preserving the basic right of keeping one’s opinions private.

## Introduction

Our increased reliance on the Internet is making it almost impossible to go about our daily lives without leaving digital traces. This datafication process is enabling corporations to scrutinize our activities at an unprecedented scale, especially given the recent breakthroughs in machine learning and big data analysis (1). Worse still, predictive modeling allows such corporations to infer private information that was never even part of our digital trace (2). For example, it has been shown that by knowing only a few “likes” that a person gives on Facebook, an algorithm can judge their personality better than their own spouse (3), and can infer various attributes including their age, gender, ethnicity, intelligence, relationship status, satisfaction with life, substance use, and sexual orientation (4). Such attribute inference can take place without people’s consent or knowledge, as demonstrated by the scandal of Cambridge Analytica, which used Facebook data to profile millions of people for political purposes (5). Profiling individuals is carried out not only by corporations, but also by governmental entities. This can be done at a massive scale, as evidenced by the mass surveillance in China, which involves the use of facial recognition algorithms as part of its controversial social credit system (6). Many fear that such a system may evolve, giving rise to an Orwellian society (7)—a dystopia where people live under constant surveillance, and AI acts as the all-knowing and omnipresent Big Brother (8). Unlike the problem of collecting personal identifiable information and sensitive data (related to specific topics such as religion, politics, sexual orientation, etc.), which can be mitigated by data protection laws such as those introduced in Europe (9) and California (10), to date no laws have been put in place to mitigate the privacy concerns stemming from attribute inference, which involves extracting private information from publicly available data that people share willingly online.

Most of the literature focuses on developing sophisticated attribute inference algorithms (2–4), leaving the individual’s privacy ever more exposed to intrusion. Here, we take the perspective of the individual who wishes to safeguard their private information from attribute inference, and argue that such an individual is not helpless in the face of prying algorithms. Specifically, we consider the possibility of deliberately modifying one’s publicly available data in the hope of making such algorithms unable to infer one’s private opinions. With this in mind, we focus on three broad research questions: firstly, *to which extent do people feel the need to hide from AI?* Secondly, *are people capable of hiding from AI without any assistance?* Finally, *can algorithms guide people through the process of hiding from AI?*

We examine these questions in the context of AI designed specifically to detect the *stance* of Twitter users toward a given topic. Here, stance is interpreted as a person’s attitude, feeling, judgment, or commitment toward the topic (11). Numerous algorithms have recently been developed to expose Twitter users’ stance toward an increasing range of topics, without paying attention to the ethical implications involved, such as violating one’s fundamental right to keep their opinion private (12). For instance, such algorithms have been used to detect people’s opinion of different candidates in the 2016 US presidential election (13). Privacy violation via stance detection can be even more problematic when the topics in question are controversial, such as one’s attitude toward immigration in the UK (14), toward refugees in the European Union (15), and toward Muslims after the 2016 ISIS attacks on Paris (16,17). Other sensitive topics that have been considered in the stance detection literature include gun control and Obamacare (18), as well as abortion, atheism, feminism, and climate change (12,19). The growing number of topics that can be analyzed by stance detection algorithms are alarming, as it suggest that people may lose the ability to conceal their opinions regarding various aspects of life, including ethics, politics, and religion.

Against this background, we investigate the aforementioned research questions in the context of stance detection on Twitter. Firstly, *to which degree do Twitter users feel the need to avoid revealing their stance*? The answer to this question would help us understand the extent of the privacy invasion issue caused by stance detection algorithms. Secondly, *how effective are people in identifying Twitter usage patterns that reveal one’s stance to a state-of-the-art machine learning algorithm*? This would help us assess people’s ability to strategically modify their usage patterns in order to conceal their own stance from that algorithm. Finally, *how effective can people be if they sought guidance from AI to modify their profile in the hope of evading stance detection algorithms?* Developing such an AI may help Twitter users protect their privacy even if their own intuition is insufficient to accomplish this task, and may prevent them from accidentally giving the wrong impression about their own stance. More broadly, such AI would empower people to take control over their public persona.

## Results

To evaluate the degree to which Twitter users feel the need to keep their stance private, we surveyed 1,143 participants recruited through Amazon Mechanical Turk. In order to be eligible, respondents had to be at least 18-y-old, live in the United States, and have a Twitter account for at least 1 y. Our survey focused on three topics that are of social, political, and religious nature, and are widely studied in the literature (12,19,20), namely: feminism, Hillary Clinton, and atheism. For each of these topics, participants were asked to indicate their stance as either “Strongly against,” “Against,” “Neither,” “In favor,” or “Strongly in favor.” Moreover, for each topic, participants were asked to specify the degree to which they feel the need to avoid revealing their stance on Twitter. To this end, following the recommendation of Leung (21), we used an 11-point Likert scale, where 0 is labeled “*I want to reveal my stance*,” and 10 is labeled “*I strongly want to keep my stance private*.” The details of the survey are provided in Appendix S1.

**Fig. 1.**
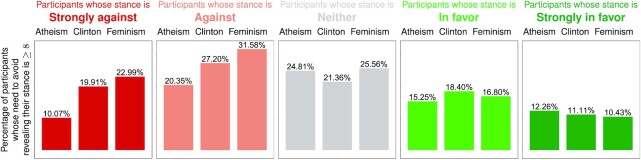
The degree to which Twitter users feel the need to avoid revealing their stance. For every topic (feminism, Hilary Clinton, and atheism), and every stance (strongly against, against, neither, in favor, and strongly in favor), participants indicated on a Likert scale from 0 to 10 the degree to which they feel the need to avoid revealing their stance on Twitter. The bar height represents the proportion of participants whose reported degree is ≥8. For instance, out of those strongly against feminism, 22.99% selected 8 or above when indicating the degree to which they feel the need to avoid revealing that stance on Twitter.

Figure 1 presents the proportion of participants who reported 8 or above on the Likert scale when assessing their need to avoid revealing their stance on Twitter (the histograms of all answers are presented in Figure S1 in Appendix S2). Interestingly, this proportion is smaller for those with an extreme stance toward a topic (be it strongly against or strongly in favor) compared to those with more moderate views. For example, when it comes to the atheism topic, $10.07\%$ and $12.26\%$ of people with extreme stance report their need to avoid revealing it as 8 or above (for the strongly against and strongly in favor stances, respectively), whereas for people with more moderate views these values are $20.35\%$, $24.81\%$, and $15.25\%$ (for the against, neither, and in favor stances, respectively). This suggests that the privacy concerns raised by stance detection algorithms are most relevant not to partisans and zealots, but rather to people with less extreme opinions. Another interesting observation is that participants who are against a topic usually feel a greater need to keep their opinion private, compared to those who are in favor of a topic (the proportion reported in Figure 1 is greater for the “Strongly against” and “Against” stances, compared to the “Strongly in favor” and “In favor” stances, with the only exception being participants who are strongly against atheism). More importantly, regardless of the topic and the stance, a considerable proportion of participants reported 8 or above on the Likert scale (10% to 23% for those with strong opinions, and 15% to 32% for the remaining participants). These findings imply that many Twitter users would rather not have their stance exposed by anyone, which is particularly alarming given the expanding repertoire of algorithms designed precisely for this purpose.

Next, we evaluate people’s ability to identify Twitter usage patterns that reveal one’s stance to an AI algorithm. To this end, we analyzed a dataset consisting of Twitter users, their tweets, their contacts (i.e., the Twitter accounts they follow), their interactions (i.e., the Twitter accounts and the websites mentioned in their tweets), and their stance toward one of the three topics mentioned earlier (i.e., feminism, Hilary Clinton, and atheism). Using this dataset, we trained a support vector machine (SVM) model, which classifies the stance of Twitter users toward a topic as either “in favor” or “against”; see the “Methods” section for more details. This classifier has been shown to provide state-of-the-art performance in stance detection (12). Our survey focuses on three types of features used by the SVM classifier: (1) a word used by the user in a tweet, (2) an account followed by the user, and (3) an account mentioned by the user in a tweet. For each topic and feature type, we identified the three features most strongly associated with the “against” stance, and the three most strongly associated with the “in favor” stance, according to the SVM classifier. For each of these features, we asked participants to specify the stance that it indicates toward the topic, where stance ranges from “Strongly against” to “Strongly in favor”. It should be noted that this is a more challenging task than simply specifying whether or not a given feature indicates *any* stance toward the topic. For example, if a person follows Hilary Clinton’s Twitter account, it suggests that they have an opinion toward her, but does not provide any indication as to whether their opinion is positive or negative. However, since our goal is not to hide whether one has an opinion, but whether that opinion is positive or negative, our experiment focuses on the more challenging task. It is also worth noting that machine learning algorithms determine their decision based on a wide variety of features, and a single word or a single account followed will likely not be decisive to such an algorithm. However, due to the impracticality of asking participants to evaluate hundreds, if not thousands, of features, we focus our attention on just a few features that are the most indicative from the algorithm’s perspective.

**Fig. 2.**
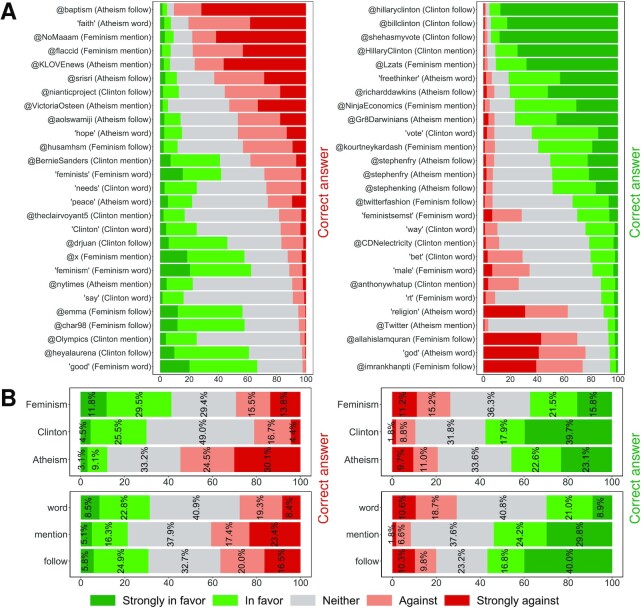
Participants’ ability to identify the stance indicated by different features. For every topic (feminism, Hillary Clinton, and atheism) and every feature type (word used by the user in a tweet, account followed by the user, and account mentioned by the user in a tweet), we selected the three features most indicative of being “against” the topic according to the SVM classifier, as well as the three features most indicative of being “in favor” of the topic. For each such feature, participants were asked to specify the stance it indicates toward the topic, where stance ranges from “strongly against” to “strongly in favor”. (A) Distribution of the participants’ classifications of the features that indicate “against” (left column) and the features that indicate “in favor” (right column). The label of each row starts with the feature, followed by the topic, and then the feature type, e.g., the row labeled “@baptism (Atheism follow)” corresponds to the results when participants are asked to classify the stance toward atheism as indicated by following the Twitter account @baptism. Rows are sorted based on the percentage of responses that are correct, i.e., those that classify the feature as “against” or “strongly against” in the left column, or classify the feature as “in favor” or “strongly in favor” in the right column. (B) The results from (A) aggregated based on the topic (upper row) and based on the feature type (lower row).

Figure 2(A) presents the distribution of responses, with the left and right columns corresponding to the features associated with the “against” and the “in favor” stances, respectively (for the exact number, see Tables S1 and S2 in Appendix S2). As can be seen, out of the 54 features included in the survey, only 20 were correctly classified by more than half of the participants, i.e., classified as “against” or “strongly against” in the left column, or as “in favor” or “strongly in favor” in the right column. Broadly similar trends were observed when omitting the participants who had neutral opinions about the topic in question (Figure S2 in Appendix S3), when considering only those who had strong opinions (Figure S3 in Appendix S3), and when considering only those who reported 8 or above when assessing their need to avoid revealing their stance on Twitter (Figure S4 in Appendix S3). Figure 2(B) aggregates the results from Figure 2(A) based on the topic and feature type under consideration. Interestingly, for all feature types, and all topics other than atheism, participants were less capable of identifying the features associated with the “against” stance than those associated with the “in favor” stance. This suggests that people who are against a topic are less capable of understanding what reveals their opinion to an AI algorithm, which is alarming since those are precisely the people who feel a greater need to hide their opinion, as we have already seen in Figure 1(B). More importantly, taking all features into consideration (regardless of whether they are associated with “against” or “in favor”), the percentage of participants who correctly classify the features for feminism, Clinton, and atheism was only 33%, 39%, and 50%, respectively. These findings suggest that people cannot reliably identify the features that reveal opinions to machine learning algorithms.

Instead of relying on human intuition to conceal their opinion, people may consider *fighting AI with AI*, i.e., receiving advice from an automated assistant on how to modify their Twitter profiles in order to hide their opinions from stance detection algorithms. Such an assistant would have to solve what is essentially an optimization problem whereby, given a “budget” specifying the maximum number of modifications that the “evader” is willing to make to their Twitter account, the goal is to identify the modifications that would optimally hide the evader’s opinion. In our complexity analysis, we focus on “*k*-nearest neighbors” as the stance detection algorithm from which the evader wishes to hide. We choose this algorithm not only due to its popularity as a general-purpose machine learning technique, but also due to its closed-form formulation, which makes it amenable to theoretical analysis. Despite the simplicity of this algorithm, our theoretical analysis shows that hiding opinions from it is at least as hard as any nondeterministic polynomial time problem, implying that no known algorithm can solve it in polynomial time; see Theorem S1 and Figure S5 in Appendix S4.

These findings imply that it is hopeless to seek an optimal way to modify the Twitter profile of a user in order to hide their opinion. Instead, the automated assistant should focus on identifying effective (albeit not optimal) modifications. To this end, we propose two heuristics. The first hides the user’s stance toward a given topic by removing from the user’s profile the features that are most strongly indicative of their stance; we refer to this heuristic as *H*^−^. The second heuristic hides the user’s stance by adding features that indicate the opposite stance; we refer to this heuristic as *H*^+^. For example, if a user is running *H*^+^ to hide the fact that they are against feminism, the heuristic could recommend following certain accounts that support the feminism movement. Note that *H*^+^ and *H*^−^ are generic, and do not specify how to select the features that will be added or removed. In our evaluation, the selection is based on the feature coefficients in SVM—a classification technique that provides state-of-the-art performance in stance detection (12) (note that this is the same technique we used to choose the features that were presented to our survey participants). We refer to the resulting heuristics as $H_{\mathit {SVM}}^+$ and $H_{\mathit {SVM}}^-$.

When evaluating these heuristics, it is important to note that the evader does not know the stance detection algorithm(s) they are hiding from. Nevertheless, even without this knowledge, the user may still hope to hide their stance by modifying their Twitter profile based on insights drawn from a state-of-the-art SVM classifier. Based on this observation, we evaluate the effectiveness of our heuristics against a number of stance detection algorithms, including the SVM classifier itself, since it can still be used by others to detect the evader’s stance. More specifically, the four stance detection algorithms that we benchmark our heuristics against are SVM (22), logistic regression (23), naive Bayes (NB) (24), and convolutional neural network (CNN) (25); see the “Methods” section for more details. Throughout our experiments, we use the SemEval dataset while focusing on the Twitter users whose stance is specified as either “in favor” or “against” one of the following five topics: feminism, Hilary Clinton, atheism, climate change, and abortion. Before evaluating our heuristics, we evaluated the stance detection algorithms themselves, to get an idea of their performance before any modifications are made to the users’ profiles. The results are summarized in Table S3 in Appendix S2, where performance is measured using the F1 score—a standard and widely used measure in the machine learning community. As can be seen, classical machine learning algorithms, namely SVM, logistic regression, and NB, outperform the more advanced deep learning alternative, namely, the CNN; these results align with previous findings from the literature (12). Another important aspect to be considered when evaluating the heuristics is who they are trying to hide from. In particular, two distinct interpretations can be considered: (i) when the stance detection is performed by the platform owner; and (ii) when it is performed by an outside party. The former has access not only to the complete set of features that can be used by a machine learning algorithm, but also to personally identifiable information of the users, such as email addresses or phone numbers. In contrast, an outside party may have access to a limited subset of the features, e.g., only those that are publicly visible. In our evaluation, we do not use any personally identifiable information. Hence, our results capture the latter scenario, but also shed light on the former one, providing a lower bound on the platform owner’s ability. It should also be noted that if we interpret the removal of features by the *H*^−^ heuristic as a preemptive action, i.e., as refraining from posting certain content, rather than deleting it after it has been posted, the heuristic will be effective even when executed against the platform owner who has access to the user’s action history. Finally, it is worth noting that our heuristics will be primarily useful to people who wish to use their social media accounts to communicate with others under their own name while being concerned about the privacy of some of their opinions. Otherwise, a much simpler solution would be to create a separate, anonymous account, or even multiple accounts—a practice known in the literature as the Sybil attack (26). Nevertheless, preserving a completely anonymous identity comes at a significant cost, e.g., it prevents users from socializing with people they know in real life. Our heuristics are intended for users who prefer to maintain their own identity when using social media.

**Fig. 3.**
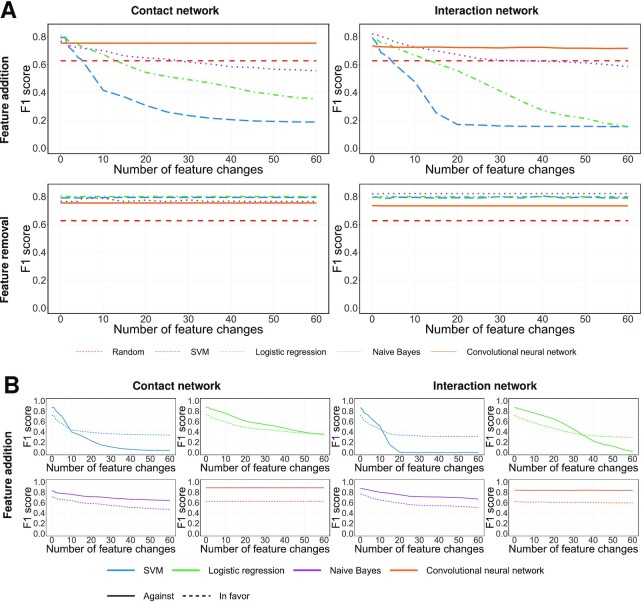
Effectiveness of stance obfuscation following the recommendation of the SVM classifier. For every person whose stance toward any of the five topics (feminism, Hilary Clinton, atheism, climate change, and abortion) is specified as either “in favor” or “against” in the SemEval dataset, we changed their features according to the feature coefficients in the SVM classifier. This was done by either using $H_{\mathit {SVM}}^-$, which removes the features that are most indicative of the person’s stance toward the topic (bottom row), or by using $H_{\mathit {SVM}}^+$, which adds the features that are most indicative of the opposite stance toward the topic (upper row). Two versions of this experiment were carried out; the first involved only the features related to the *contact network*, i.e., the accounts followed by the person; whereas the second involved only the features related to the *interaction network*, i.e., the accounts and domains mentioned in the person’s tweets. The left column presents the results of the first version of the experiment, whereas the right column presents those of the second version. We plot the F1 score of different stance detection algorithms as a function of the number of modifications made by our heuristics. Here, lower F1 scores indicate better obfuscation of the person’s stance. (A) Results for feature addition and feature removal, taking all users into consideration. (B) Results for feature addition, where users are disaggregated into those who are in favor of the topic, and those who are against it.

We evaluated our heuristics when the features being modified, i.e., added by $H_{\mathit {SVM}}^+$ or removed by $H_{\mathit {SVM}}^-$, are related to either the user’s contacts (i.e., the Twitter accounts they follow) or the user’s interactions (i.e., the Twitter accounts and the websites mentioned in their tweets). Note that the stance detection algorithms are trained on the original dataset rather than the modified dataset. To put it differently, we train them before, not after, the features are modified using our heuristics. It should also be noted that these experiments evaluate the impact of our heuristic on a given topic, without considering the possible side-effect of accidentally influencing one’s perceived stance toward the other two topics; for an analysis of such cross-topic implications, see Figure S6 in Appendix S5. Figure 3(A) shows how our heuristics affect the performance of different stance detection algorithms on a given topic. As a baseline, we also depict the performance of a random classifier whereby the probability of an individual being against a given topic is simply equal to the proportion of individuals who are against that topic in the training set. Note that the performance of this baseline is not affected by our heuristics, since it does not take any features into consideration. As a measure of performance, we plot the F1 score, where lower values indicate better obfuscation of the person’s stance. As can be seen in Figure 3(A), the type of the features being modified—be it related to the contact network or to the interaction network—does not seem to play a crucial role in the effectiveness of the heuristic. This implies that people can choose the type of features to modify based on their own preferences, without worrying about compromising the process of hiding their opinion. In contrast, the choice of adding vs. removing features seems to be critical. Specifically, the removal of features has a negligible effect on three out the four stance detection algorithms. On the other hand, the addition of features is much more effective, to the point of dropping the performance of three out of the four algorithms below the random baseline. This difference in effectiveness between adding and removing features is likely caused by the fact that, after the feature modification, the users would still have other features in their profile that indicate their true stance. Thus, the removal of features would remove some (but not all) of the evidence indicating one’s stance. On the other hand, the addition of features would plant evidence indicating the opposite stance, thereby confusing the classifiers. It is worth noting that the removal-based heuristic would render social media less valuable to people, as they would no longer be able to follow the Twitter accounts they were interested in. In contrast, the addition-based heuristic is more viable as it lets people follow the same Twitter accounts as before, albeit with an additional noise. It should be noted that the CNN, which initially has the worst performance compared to other stance detection algorithms (as shown in Table S3 in Appendix S2) is the most resilient to our heuristic, highlighting the potential risk of developing deep learning algorithms that are even more challenging to hide from. This significant level of resilience is likely due to the fact that the neural networks operate on high-dimensional vectors, and the outcome of their computations cannot be easily altered by modifying only a few data points.

Next, we compare the impact of our heuristic on the users who are in favor of the topic vs. those who are against it. Here, we restrict our attention to feature addition, since it has already been shown to be more effective than feature removal. The results are depicted in Figure 3(B). Broadly speaking, the heuristic seems more effective for those who are against the topic of interest. This matters because those are precisely the users who feel a greater need to hide (as we have shown in Figure 1) and are also the ones who are less capable of identifying the features that expose one’s opinion (as we have shown in Figure 2). In other words, the heuristic seems more effective for the users who need it the most. Finally, our finding—that the heuristic can hide the stance against a topic more effectively than in favor of that topic—has an intriguing implication: instead of trying to hide being in favor of a topic, it might be more effective to hide being against the “opposite” of that topic, e.g., hide being against religion instead of being in favor of atheism. Evaluating the effectiveness of such an approach is not possible given the SemEval dataset, since it does not include any data pertaining to topics that can be thought of as opposites to the ones considered in our study. As such, evaluating this approach is out of the scope of our study, but seems to be an interesting future direction.

## Discussion

Recent developments in stance detection algorithms are a warning call, showing that the basic right to keep our opinions private can no longer be taken for granted. We sought to understand the extent of this problem in the context of Twitter. Altogether, our findings suggest that people’s online activities may reveal their personal beliefs and opinions to AI without them realizing it. Worse still, even if they make that realization, their ability to hide from AI seems unreliable, especially if stance detection algorithms continue to evolve, scrutinizing ever more subtle cues in our behavior. Perhaps a more practical approach is to “fight AI with AI,” i.e., use an automated assistant that would bring to our attention the activities that may expose our opinions to AI. To investigate this possibility, we analyzed the optimization problem faced by such an automated assistant when determining the most effective modifications to one’s user profile. Our empirical evaluations demonstrated that the addition of features indicating the opposite opinion can be effective against a number of stance detection algorithms.

Our study comes with a number of limitations. First, our study focuses on just three topics, which serve as examples demonstrating the need to hide opinions about important social, political, and religious issues, and demonstrating people’s inability to effectively identify features that reveal their opinions to AI. Nevertheless, these topics clearly do not cover the entire spectrum of sensitive issues, and more research is needed to cover a wider range of topics. Second, our sample is reasonably large (with data from 1,143 individuals) but not representative, since MTurk workers tend to be younger, more educated, and more technologically savvy than the average American (27). Relying on MTurk has another limitation: although we ensured that all our participants have been Twitter users for at least 1 y, their judgement of various Twitter accounts is limited compared to users who actually follow those accounts. Thus, future studies are needed to evaluate the problem of hiding opinions “in the field.” Another limitation of our approach is the potential risk of affecting one’s stance toward other topics that are not considered in the calculations. Our experiments suggest that there is a tradeoff between hiding one’s opinion toward a topic and affecting one’s perceived opinion toward other topics. They also suggest that, as long as the hiding efforts are not excessive, then the cross-topic influence is likely to be limited. Nevertheless, it remains unknown whether the same holds for *any* two topics, especially if the two are strongly related. For instance, hiding one’s opinion about a political candidate may accidentally expose their opinion about immigration. Such cross-topic implications need to be examined and evaluated more thoroughly, taking a wider range of topics into consideration. Yet another limitation is that all of our hiding methods were tested against stance detection algorithms, but not against humans. In other words, while we have shown that our heuristics can mislead a variety of algorithms, their effect on the evader’s stance as perceived by fellow social media users remains unknown. The findings presented in Figure 2 suggest that people infer each other’s stance in a significantly different way than machine learning algorithms. Consequently, one might speculate that our heuristics would not change the evader’s opinion as perceived by other users. On one hand, this can be thought of as a drawback, as hiding opinions from people would require a completely different portfolio of techniques. On the other hand, a user who is only interested in misleading algorithms may not run the risk of giving an unfavorable impression to other users. Perhaps a hybrid approach would overcome these issues, with the algorithm making suggestions, and the user having the ultimate decision to decline or approve these suggestions as they see fit. Finally, it should be noted that, when using our heuristic to hide one’s stance toward a topic, their opinion does not disappear but rather changes. For instance, hiding the fact that one is proatheism would make the classifiers conclude that they are *not* proatheism, rather than concluding that they are against atheism, or that their opinion toward atheism is neutral. To some users, making the stance appear neutral might be more desirable than flipping the direction of their perceived stance. However, this would be impractical since most stance detection classifiers are binary, i.e., they classify users’ stance as either in favor or against (28), and are thus incapable of producing a neutral class. Consequently, our heuristics should be used by people who wish to flip their perceived stance rather than make it appear as neutral, and they should be made aware of the distinction between the two.

Unlike our study, where the responsibility of protecting one’s privacy lies in one’s hands, the majority of the literature on privacy preservation assumes that this responsibility lies with a central authority (29–33). However, this kind of approach is only useful if it is effectively enforced, which appears to be rather challenging in the world of social media, as evidenced by a number of scandals that involved violating the privacy of social media users (5,34,35). In an attempt to address the shortcomings of centralized privacy preservation approaches, a number of solutions have recently been proposed to empower the people whose privacy is being violated, and put the control in their hands rather than in the hands of a central authority (36–40). Nevertheless, none of the existing solutions can be used to hide the opinions of Twitter users from stance detection algorithms. The literature that is perhaps the most relevant to our work is that of adversarial machine learning (41,42), which attempts to mislead machine learning models via doctored input. In particular, our study is strongly related to the literature concerned with adversarial attack on networks (43), in which the attacker introduces network modifications to disrupt node classification (44–46). Just like the literature on adversarial machine learning, our study also aims to strategically manipulate the input of an algorithm in order to influence its output. However, none of the existing techniques in this literature can be readily applied to hide the opinions of Twitter users from stance detection algorithms. Our study is also relevant to the literature looking at personal attitudes to privacy (47). In this literature, one phenomenon that is relevant to our study is the privacy paradox (48,49), where people’s attitude toward privacy is inconsistent with their behavior. For instance, although social media users tend to value their privacy, they share countless posts that compromise it (50). The way in which people assess this tradeoff—between preserving one’s privacy and enjoying certain benefits—has been studied in the literature on the privacy calculus (51,52). In this context, our study suggests that assessing this tradeoff is harder in the age of AI, since people do not fully understand the relationship between what they post online and how their opinion is classified by machine learning algorithms. Finally, we mention the literature on solutions that protect the user’s personal identifiable information, either by adding noise to it (53,54), or by removing it entirely from the user’s digital trace (55). In principle, these solutions resemble ours, since we also add or remove features from the user’s profile. However, unlike ours, these solutions do not provide recommendations regarding what information the users should share on social media platforms, e.g., they do not recommend removing certain keywords from one’s tweet, or following certain Twitter accounts.

If our hiding methods were to be implemented, a number of practical considerations have to be made. First, our methods require determining the coefficients of an SVM model, which is clearly too complex to be implemented by a member of the general public. For this solution to be practical, it has to be implemented by a third party and made readily available to Twitter users, possibly as a plugin or an add-on. Second, as stance detection algorithms continue to improve and evolve, such an AI assistant would have to be regularly updated, as is the case with antivirus software. Third, such an AI assistant should maintain a portfolio of topics, and should report to the user how their opinion regarding each such topic appears in the eyes of stance detection algorithms. This way, if an activity exposes a certain opinion of the user without them realizing it, the assistant may warn the user about the unintended consequences of this activity. Such a feature would be even more critical in situations where the user unintentionally performs activities that give the wrong impression about their opinions toward sensitive topics. For instance, if a person realizes that a certain tweet would mistakenly make them appear racist in the eyes of stance detection algorithms, then an early warning system may prevent the user from posting such a tweet to start with. In the future, such an assistant may even evolve beyond the context of privacy preservation, by allowing the user to not only conceal their private information, but also determine how they portray themselves to the ever-watchful eyes of AI observers, and to the world as a whole. Fourth, implementing our hiding methods might have side effects that are difficult to predict based solely on simulations. For example, it might happen that removing some network connections from the platform will facilitate the creation of filter bubbles or reduce the number of bridges between communities. According to our evaluation, the more effective technique would be to connect with accounts from the other side of the spectrum, seemingly promoting diversity and bursting the bubble. Nevertheless, the final outcomes are difficult to predict with certainty based on our simulations. Developing opinion mining algorithms raises an ethical concern, as it could violate people’s basic right of choosing which of their opinions to share with the world. There is a pressing need to address these concerns, especially given the rapid advancement in AI in general and machine learning in particular. One solution could be to create robust public policies about what is allowed to be developed. However, such policies are difficult, if not impossible, to enforce, since classifiers can always be developed and used in secret. Until such solutions are implemented, people’s opinions are left exposed to AI, and our study provides an alternative pathway.

## Methods

Our analysis is based on the SemEval 2016 dataset (20). This dataset contains multiple tweets, and specifies for every tweet its ID in the Twitter database, its content, its topic (atheism, climate change, feminism, Hillary Clinton, or abortion), and the stance indicated by the tweet toward its topic (in favor, against, or neither of the two). We kept only the tweets that satisfy the following conditions: (i) they indicate the user’s stance as either “in favor” or “against”; (ii) their authors did not have their account deleted or suspended by the time of our study. Subsequently, we used Twitter’s REST API to gather information about the *contacts* and *interactions* of each Twitter user in our dataset, i.e., each person who authored a tweet in the dataset. Specifically, contact data consist of the user’s friends (i.e., the accounts followed by that user), whereas interaction data consist of both the Twitter accounts and the websites mentioned in the user’s home timeline (i.e., the tweets and retweets posted by the user). For more details about the resulting dataset, see Table S4 in Appendix S2.

For stance detection, we use four machine learning algorithms to infer the stance on the dataset, namely, SVM (22), logistic regression (23), CNN (25), and NB (24). As for SVM, we use a linear kernel with a C-value in [1,1000], where the optimal value is selected using grid search. For logistic regression, we use *”lbfgs”* with the random state being equal to zero. For the CNN, we use three layers, and compile the network using the Adam optimizer. Finally, to implement the NB algorithm, we use the Multinomial NB as the configuration for the stance detection model.

## Supplementary Material

pgac256_Supplemental_File

## Data Availability

The SemEval 2016 dataset that we used in our analysis is publicly available online (56). Anonymized data collected by us are publicly available in an online repository (57).
