# Supplementary material for: Hiding opinions from machine learning
Source: PNAS Nexus. 2022 Nov 16;1(5):pgac256. doi: 10.1093/pnasnexus/pgac256 (PMC9802261; doi:10.1093/pnasnexus/pgac256)
Supplement: pgac256_Supplemental_File [file pgac256_supplemental_file.pdf]

Supplementary materials for

Hiding opinions from machine learning

Marcin Waniek[a], Walid Magdy[b,*], and Talal Rahwan[a,*]

[a]Computer Science, New York University Abu Dhabi, Abu Dhabi, United Arab Emirates

[b]School of Informatics, The University of Edinburgh, Edinburgh, United Kingdom

[*]Joint corresponding authors. E-mails: wmagdy@inf.ed.ac.uk, talal.rahwan@nyu.edu

This document is structured as follows:

- **Appendix S1** (*page 2*) presents the survey questionnaire;
- **Appendix S2** (*page 8*) presents supplementary tables and figures;
- **Appendix S3** (*page 11*) presents robustness checks;
- **Appendix S4** (*page 14*) presents the mathematical proof of our theoretical result;
- **Appendix S5** (*page 17*) presents evaluation of the cross-topic implications of hiding.

S1 Survey Questionnaire and Additional Results

To evaluate the degree to which Twitter users feel the need to keep their stance private, we surveyed 1,143 participants recruited through Amazon Mechanical Turk. In order to be eligible, respondents had to be at least 18 years old, live in the US, and have a Twitter account for at least one year.

S1.1 Consent Form

Welcome to this study exploring the people's perception towards online signals that reveal their stance when using Twitter. You are eligible to participate in the study at this time if you are:

- *18 years of age or older;*
- *Live in the United States of America (USA);*
- *Have a Twitter account for more than 1 year.*

The goal of this study is to discover how the user of social media platform identify their support/opposing stance towards a topic using social media online factors. By "stance" we mean your "viewpoint", or your "attitude" towards a person or a topic .

What will happen if I decide to take part?

You will be asked to fill out an online survey about the online factors that indicates stance towards a topic. You will also be asked to provide basic demographic information and prior experience with social media. The questionnaire should take approximately 10 minutes to complete.

Do I have to take part?

No, participation in this study is entirely up to you. You can withdraw from the study at any time, without giving a reason. Your rights will not be affected. If you wish to withdraw, contact the PI. We will stop using your data in any publications or presentations submitted after you have withdrawn consent. However, we will keep copies of your original consent, and of your withdrawal request.

Compensation and benefits

An amount of $2.50 will be paid upon successful completion of the survey.

Are there any risks associated with taking part?

There are no significant risks associated with participation.

What will happen to the results of this study?

The results of this study may be summarized in published articles, reports and presentations. Quotes or key findings will be anonymized: we will remove any information that could, in our assessment, allow anyone to identify you. Information can also be used for similar future research. Your data may be archived for a minimum of 3 years.

Data protection and confidentiality

Your data will be processed in accordance with Data Protection Law. All information collected about you will be kept strictly confidential. Your data will be referred to by a unique participant number rather than by name or platform ID.

All electronic data will be stored on password-protected encrypted computers, on the School of Informatics' secure file servers, or on the University's secure encrypted cloud storage services (DataShare, DataSync, or Sharepoint).

Who is conducting this research and who can I contact about it?

This research is conducted by a team of researchers at the University of Edinburgh and New York University Abu Dhabi, the members of this research are Abeer Aldayel, Walid Magdy, Talal Rahwan, and Marcin Waniek. The research has been approved by the respective Institutional Review Boards.

For questions about the rights of research participants, you may contact the Institutional Review Boards Committee, New York University Abu Dhabi, irbnyuad@nyu.edu.

If you have any questions, suggestions or concerns, please feel free to reach out to us at a.aldayel@ed.ac.uk an email address that only researchers associated with this project have access to.

Please do not complete the survey more than once. Upon finishing the survey you will receive a completion code. The payment of $2.50 will be made once you've entered that code in the space provided. Please do not close the browser with your MTurk account.

By continuing you agree that:

You have read the above information and agree to participate in the study.

S1.2 Demographic Questions

1. ***What is your worker ID?***

   [empty field to be filled by a number]

2. ***What is your state of residence?***

   [drop-down list to choose state]

3. ***What is the sex listed on your birth certificate ?***

   (a) *Male*
   (b) *Female*
   (c) *I prefer not to say*

4. ***What is your ethnicity?***

   (a) *Hispanic*
   (b) *Non-Hispanic*

5. ***What is your race?***

   (a) *White*
   (b) *Black or African American*
   (c) *Asian*
   (d) *Native American*
   (e) *Middle Eastern or North African*
   (f) *Mixed*
   (g) *Other* [empty field to be filled by text]

6. ***What is your age?***

   [empty field to be filled by a number]

7. ***What is your highest completed level of education?***

   (a) *Less than high school*
   (b) *High school graduate or equivalent (e.g., GED)*
   (c) *Some college*
   (d) *2 year degree (i.e. Associate's degree)*
   (e) *4 year degree (i.e. Bachelor's degree)*
   (f) *Masters or Professional degree (i.e. MBA, MPP, etc)*

(g) *Doctoral Degree*

8. ***What best describes your employment situation?***

   (a) *Full-time employed*
   (b) *Part-time employed*
   (c) *Unemployed*
   (d) *Caregiver (e.g., children, elderly) or homemaker*
   (e) *Retired*
   (f) *Full-time student*
   (g) *Other* [empty field to be filled by text]

9. ***What was your yearly personal income in 2019 (include salary, interests, returns on investments, etc)?***

   (a) *Less than $10,000*
   (b) *$10,000-$19,999*
   (c) *$20,000-$29,999*
   (d) *$30,000-$39,999*
   (e) *$40,000-$49,999*
   (f) *$50,000-$59,999*
   (g) *$60,000-$69,999*
   (h) *$70,000-$79,999*
   (i) *$80,000-$99,999*
   (j) *$100,000-$119,999*
   (k) *$120,000-$149,999*
   (l) *$150,000-$199,999*
   (m) *$200,000 - more*

S1.3 Topic 1: Hillary Clinton

1. ***What is your stance toward Hillary Clinton?***

   (a) *Strongly against*
   (b) *Against*
   (c) *Neither*
   (d) *In favor*
   (e) *Strongly in favor*

2. ***If a person is using one of the below words in a tweet, what would you assume is the stance of that person towards Hillary Clinton?***

   [Next to every word is a group of radio buttons with options “Strongly against”, “Against”, “Neither”, “In favor”, and “Strongly in favor”]

   (a) *Clinton*
   (b) *say*
   (c) *needs*
   (d) *vote*
   (e) *way*
   (f) *bet*

3. ***If a person is following one of the below accounts, what would you assume is the stance of that person towards Hilary Clinton?***

   [Next to every account is a group of radio buttons with options “Strongly against”, “Against”, “Neither”, “In favor”, and “Strongly in favor”]

   (a) *@heyalaurena: Explorer of the world*

   (b) *@nianticproject: A slow leak of information is coming my way. Visions – affecting people, strange occurrences, secrets and lies. I'm watching, piecing things together*
   (c) *@drjuan: neuroscientist, teacher, raconteur, bon vivant, rapscallion*
   (d) *@shehasmyvote: Hillary Rodham Clinton for President 2016*
   (e) *@billclinton: Father. Grandfather. 42nd President of the United States. Founder, Clinton Foundation.*
   (f) *@hillaryclinton: 2016 Democratic Nominee, SecState, Senator, hair icon. Mom, Wife, Grandma x2, lawyer, advocate, fan of walks in the woods & standing up for our democracy*

4. ***If a person posted a tweet that mentions one of the below accounts, what would you assume is the stance of that person towards Hilary Clinton?***

   [Next to every account is a group of radio buttons with options "Strongly against", "Against", "Neither", "In favor", and "Strongly in favor"]

   (a) *@BernieSanders: U.S. Senator from Vermont and candidate for President of the United States*
   (b) *@theclairvoyant5*
   (c) *@Olympics: The Olympic Games - friendship, respect, and excellence*
   (d) *@CDNelectricity: We are the Canadian Electricity Association (CEA) The voice of electricity generation, transmission and distribution in Canada*
   (e) *@anthonywhatup*
   (f) *@HillaryClinton:2016 Democratic Nominee, SecState, Senator, hair icon. Mom, Wife, Grandma x3, lawyer, advocate, fan of walks in the woods & standing up for our democracy*

S1.4 Topic 2: Feminist Movement

1. ***What is your stance toward Feminist Movement?***

   (a) *Strongly against*
   (b) *Against*
   (c) *Neither*
   (d) *In favor*
   (e) *Strongly in favor*

2. ***If a person is using one of the below words in a tweet, what would you assume is the stance of that person towards Feminist Movement?***

   [Next to every word is a group of radio buttons with options "Strongly against", "Against", "Neither", "In favor", and "Strongly in favor"]

   (a) *feminists*
   (b) *feminism*
   (c) *good*
   (d) *male*
   (e) *rt*
   (f) *feminist semst*

3. ***If a person is following one of the below accounts, what would you assume is the stance of that person towards Feminist Movement?***

   [Next to every account is a group of radio buttons with options "Strongly against", "Against", "Neither", "In favor", and "Strongly in favor"]

   (a) *@char98_x: A&N*
   (b) *@emma_nicole246: 717*
   (c) *@husamhsm: Alfredo tweeted me 'hi' the next day justin followed me holy shit — justin followed December 16th 2013*
   (d) *@twitterfashion:Twitter, but make it fashion*

(e) *@allahislamquran: #Allah #Islam #Quran One and Only ♥* [the sentence "*in the name of God*", inserted here in Arabic] *♥ & ♥ Prophets* [the sentence "*peace be upon him*", inserted here in Arabic] *♥ #AllahIslamQuran*
(f) *@imrankhanpti: Prime Minister of Pakistan*

4. ***If a person posted a tweet that mentions one of the below accounts, what would you assume is the stance of that person towards Feminist Movement?***

   [Next to every account is a group of radio buttons with options "Strongly against", "Against", "Neither", "In favor", and "Strongly in favor"]

   (a) *@flaccid_joe: Fuck that bitch, this is Russia. - Big Igor*
   (b) *@x_aeon_x: Average centrist moderate independent in the middle. Between left & right. Not up or down. Horizontal. 5/10. Only my sexuality is bent. who/whom/whose*
   (c) *@NoMaaam: If you're mad its because its true. Pressing block only proves ur ignorance bc ur blocking information. #feminismisawful*
   (d) *@LZats: Literary Agent. Host@printrunpodcast w@erikhane Intersectional feminist. Geek. Beer lover/tea snob. Pibble enthusiast. She/her. Tweets my own*
   (e) *@NinjaEconomics: Clever musings and first-world complaints from a manic pixie wannabe-economist. Also, @ACLUcrime fighter (all views my own)*
   (f) *@kourtneykardash*

S1.5 Topic 3: Atheism

1. ***What is your stance toward Atheism?***

   (a) *Strongly against*
   (b) *Against*
   (c) *Neither*
   (d) *In favor*
   (e) *Strongly in favor*

2. ***If a person is using one of the below words in a tweet, what would you assume is the stance of that person towards Atheism?***

   [Next to every word is a group of radio buttons with options "Strongly against", "Against", "Neither", "In favor", and "Strongly in favor"]

   (a) *hope*
   (b) *faith*
   (c) *peace*
   (d) *god*
   (e) *religion*
   (f) *freethinker*

3. ***If a person is following one of the below accounts, what would you assume is the stance of that person towards Atheism?***

   [Next to every account is a group of radio buttons with options "Strongly against", "Against", "Neither", "In favor", and "Strongly in favor"]

   (a) *@baptism_saves: Pastor at (link: http://www.churchofgodonline.org) churchofgodonline.org and church of God @kingjamesonline. "The like figure whereunto even baptism doth also now save us: 1 Peter 3:21 KJV*
   (b) *@srisri Mission: To See a Smile on Every Face; One World Family (Vasudhaiva Kutumbakam) S̃ri Sri Ravi Shankar*
   (c) *@aolswamiji My Tweets are my personal opinions. RTs may or may not be endorsements*

(d) *@stephenfry How can I tell you what I think until I've heard what I'm going to say? (Never read DMs I'm afraid)*
(e) *@richarddawkins UK biologist & writer. Science, the poetry of reality. Good-humoured ridicule of religions. RTs don't imply endorsement, nor exhaustive research of tweeter's CV*
(f) *@stephenking Author*

4. ***If a person posted a tweet that mentions one of the below accounts, what would you assume is the stance of that person towards Atheism?***

   [Next to every account is a group of radio buttons with options "Strongly against", "Against", "Neither", "In favor", and "Strongly in favor"]

   (a) *@VictoriaOsteen*
   (b) *@nytimes: "The Weekly" is our new TV series. Episodes air Sundays at 10 p.m. on FX and on Hulu the next day.*
   (c) *@KLOVEnews: Thanks for stopping by for news + useful, encouraging and faith-based stories. Got news for us? newstip@klove.com*
   (d) *@stephenfry: How can I tell you what I think until I've heard what I'm going to say?*
   (e) *@Gr8Darwinians: Biped ape who loves science & punk rock music. Blind acceptance is a sign for stupid fools to stand in line*
   (f) *@Twitter: What's happening?!*

S1.6 The Need to Hide Stance

Indicate the degree to which you feel the need to avoid revealing your stance on Twitter towards the following topics.

1. *Hillary Clinton*

   [Likert scale with values: 0, 1, . . . , 10, where "0" is labeled as "I want to reveal my stance" and "10" is labeled as "I strongly want to keep my stance private"]

2. *Feminist Movement*

   [Likert scale with values: 0, 1, . . . , 10, where "0" is labeled as "I want to reveal my stance" and "10" is labeled as "I strongly want to keep my stance private"]

3. *Atheism*

   [Likert scale with values: 0, 1, . . . , 10, where "0" is labeled as "I want to reveal my stance" and "10" is labeled as "I strongly want to keep my stance private"]

S2 Supplementary Tables and Figures

Feature	Strongly in favor	In favor	Neither	Against	Strongly against
@baptism (Atheism follow)	1.64%	3.19%	4.63%	18.82%	71.72%
'faith' (Atheism word)	2.90%	4.63%	11.87%	42.28%	38.32%
@NoMaaam (Feminism mention)	3.28%	5.98%	12.84%	16.22%	61.68%
@flaccid (Feminism mention)	1.45%	5.79%	15.25%	34.17%	43.34%
@KLOVEnews (Atheism mention)	2.51%	4.34%	16.89%	19.79%	56.47%
@srisri (Atheism follow)	3.57%	8.01%	25.48%	34.17%	28.76%
@nianticproject (Clinton follow)	2.03%	10.81%	31.56%	37.84%	17.76%
@VictoriaOsteen (Atheism mention)	1.74%	3.76%	41.99%	19.50%	33.01%
@aolswamiji (Atheism follow)	2.99%	11.10%	39.29%	28.96%	17.66%
'hope' (Atheism word)	2.80%	12.36%	38.22%	33.40%	13.22%
@husamhsm (Feminism follow)	2.41%	9.65%	44.79%	33.88%	9.27%
@BernieSanders (Clinton mention)	7.14%	33.98%	20.75%	31.37%	6.76%
'feminists' (Feminism word)	15.54%	26.35%	29.54%	25.39%	3.19%
'needs' (Clinton word)	3.19%	22.01%	47.68%	23.75%	3.38%
'peace' (Atheism word)	5.50%	16.51%	51.83%	16.60%	9.56%
@theclairvoyant5 (Clinton mention)	2.41%	14.48%	64.67%	15.35%	3.09%
'Clinton' (Clinton word)	4.15%	21.04%	57.43%	13.51%	3.86%
@drjuan (Clinton follow)	5.98%	40.15%	37.07%	14.96%	1.83%
@x (Feminism mention)	18.53%	39.38%	29.54%	9.56%	2.99%
'feminism' (Feminism word)	20.56%	41.80%	26.16%	9.07%	2.41%
@nytimes (Atheism mention)	4.44%	17.95%	68.15%	7.43%	2.03%
'say' (Clinton word)	1.74%	14.48%	74.71%	7.82%	1.25%
@emma (Feminism follow)	12.16%	44.31%	38.32%	4.63%	0.58%
@char98 (Feminism follow)	11.97%	45.95%	37.26%	4.44%	0.39%
@Olympics (Clinton mention)	3.96%	21.24%	70.75%	3.19%	0.87%
@heyalaurena (Clinton follow)	9.75%	51.25%	36.29%	2.32%	0.39%
'good' (Feminism word)	20.27%	46.33%	31.08%	2.22%	0.10%

Table S1: Numeric values from the left column of Figure 2a in the main article. The first column contains the description of the feature, in the same format as in Figure 2. The remaining columns present the percentage of responses that identified the feature as indicating each of the five stances.

Feature	Strongly in favor	In favor	Neither	Against	Strongly against
@hillaryclinton (Clinton follow)	87.36%	8.11%	2.70%	0.97%	0.87%
@billclinton (Clinton follow)	82.34%	12.16%	3.19%	1.25%	1.06%
@shehasmyvote (Clinton follow)	88.03%	6.37%	2.70%	2.03%	0.87%
@HillaryClinton (Clinton mention)	74.71%	16.41%	6.18%	1.74%	0.97%
@Lzats (Feminism mention)	68.05%	22.78%	5.98%	2.32%	0.87%
'freethinker' (Atheism word)	43.05%	38.32%	12.36%	4.25%	2.03%
@richarddawkins (Atheism follow)	52.12%	28.38%	11.29%	5.69%	2.51%
@NinjaEconomics (Feminism mention)	31.08%	45.56%	18.73%	3.57%	1.06%
@Gr8Darwinians (Atheism mention)	45.75%	30.79%	15.25%	4.44%	3.76%
'vote' (Clinton word)	14.86%	49.13%	27.80%	5.60%	2.61%
@kourtneykardash (Feminism mention)	19.21%	40.06%	32.24%	7.34%	1.16%
@stephenfry (Atheism follow)	22.49%	27.90%	41.70%	5.98%	1.93%
@stephenfry (Atheism mention)	21.72%	27.22%	43.92%	4.73%	2.41%
@stephenking (Atheism follow)	16.51%	32.14%	44.59%	4.92%	1.83%
@twitterfashion (Feminism follow)	6.95%	26.83%	59.46%	5.69%	1.06%
'feministsemst' (Feminism word)	6.37%	23.94%	41.31%	21.91%	6.47%
'way' (Clinton word)	2.61%	21.62%	65.35%	9.27%	1.16%
@CDNelectricity (Clinton mention)	3.28%	18.15%	66.89%	9.94%	1.74%
'bet' (Clinton word)	2.22%	18.24%	50.39%	25.97%	3.19%
'male' (Feminism word)	3.38%	15.73%	46.62%	27.80%	6.47%
@anthonywhatup (Clinton mention)	2.03%	10.81%	60.91%	22.68%	3.57%
'rt' (Feminism word)	2.90%	9.65%	78.76%	6.95%	1.74%
'religion' (Atheism word)	2.61%	8.20%	26.54%	31.56%	31.08%
@Twitter (Atheism mention)	2.12%	5.60%	88.71%	2.80%	0.77%
@allahislamquran (Feminism follow)	2.41%	4.83%	22.97%	26.93%	42.86%
'god' (Atheism word)	1.93%	4.44%	17.76%	34.75%	41.12%
@imrankhanpti (Feminism follow)	1.64%	4.25%	20.46%	34.36%	39.29%

Table S2: Numeric values from the right column of Figure 2a in the main article. The first column contains the description of the feature, in the same format as in Figure 2. The remaining columns present the percentage of responses that identified the feature as indicating each of the five stances.

	Contact			Interactions		
**Prediction Model**	**In favor**	**Against**	**Average**	**In favor**	**Against**	**Average**
Random baseline	51.16	74.18	62.67	51.16	74.18	62.67
Support vector machine	71.55	86.59	79.07	72.00	86.92	79.46
Logistic regression	73.42	87.62	80.52	71.30	87.21	79.26
Naive Bayes	70.82	82.37	76.59	76.66	87.25	81.96
Convolutional neural network	62.25	88.56	75.40	62.36	84.61	73.48

Table S3: The F1 scores of stance detection algorithms before the hiding process. The table presents the F1 scores of four stance detection algorithms on the SemEval dataset, focusing on the Twitter users whose stance is specified as either "in favor" or "against" one of the following five topics: feminism, Hilary Clinton, atheism, climate change, and abortion. The algorithms use the original set of features, i.e., before tampering with it via our stance obfuscation heuristics. The features are either related to the users' contacts (i.e., the Twitter accounts they follow), or the users' interactions (i.e., the Twitter accounts and the websites mentioned in their tweets). For each type of features, the table presents the F1 score for the users whose stance is "in favor", the F1 score for those whose stance is "against", and the average of those two numbers.

Topic	Users	Home timeline tweets	Accounts (interactions)	Domains (interactions)	Friends (contacts)
Atheism	550	832773	121548	5834	375695
Climate change	461	607095	135915	8938	280536
Feminism	524	778512	165107	6384	250537
Hillary Clinton	670	1043282	208426	7111	435612
Abortion	670	924734	190848	7725	405269
Total	2875	4186396	821844	35992	1747649
Unique total	2234	4028574	721354	25647	1365075

Table S4: Detailed information about the size of our dataset. The first column contains the number of users extracted from the SemEval dataset. The second column contains the number of tweets from the home timeline of the users; these are the tweets from which the users' interactions were inferred. The third column contains the number of accounts that were mentioned by the users in their tweets. The fourth column contains the number of web domains that were mentioned by the users in their tweets. The fifth column contains the number of accounts that were followed by the users.

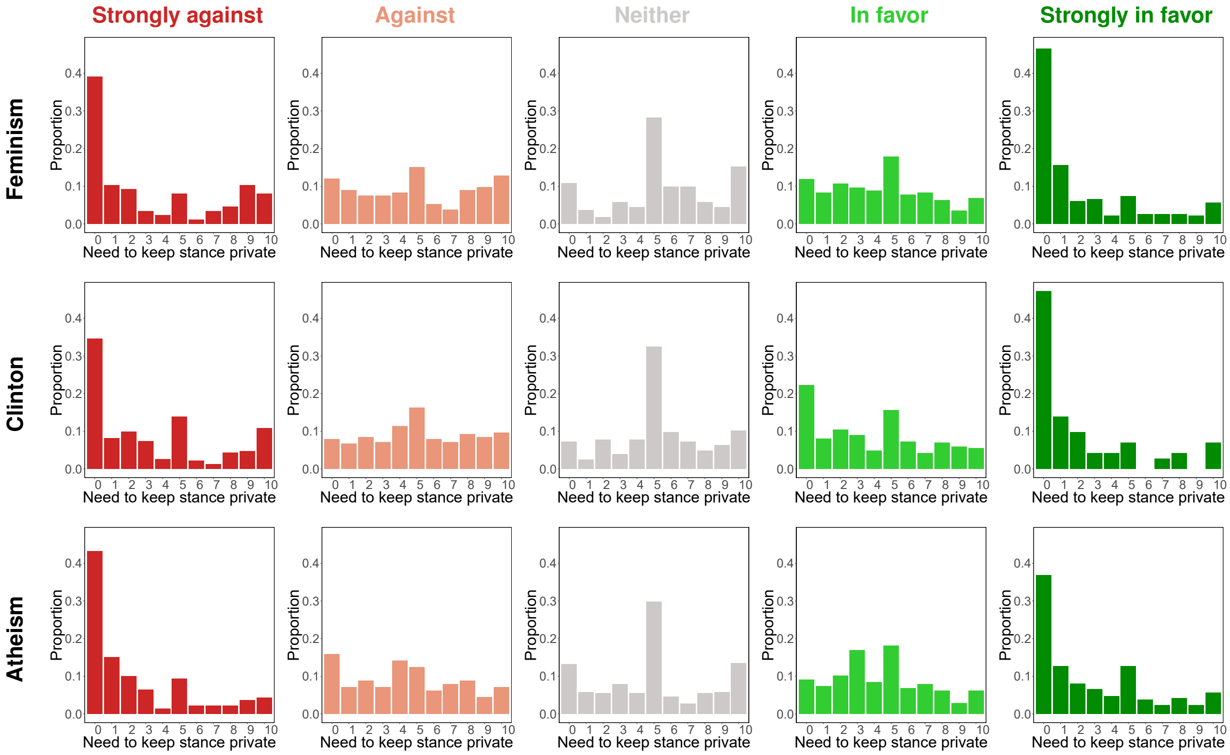

Figure S1: The distribution of the need to avoid revealing one's stance. For every topic (feminism, Hilary Clinton, and atheism), the participants indicated on a Likert scale from 0 to 10 the degree to which they feel the need to avoid revealing their stance on Twitter. For each topic, results are presented separately for each stance towards that topic, where stance ranges from "Strongly against" (dark red) to "Strongly in favor" (dark green). Each plot presents the distribution of responses for a given topic and a given stance towards that topic.

S3 Robustness Checks

When presenting Figure 2a in the main article, we noted that only 20 out of the 54 features included in our survey were correctly classified by more than half the participants. This section shows that similar trends hold when omitting the participants who had neutral opinions about the topic in question (Figure S2), when considering only those who had strong opinions (Figure S3), and when considering only those who reported 8 or above when assessing their need to avoid revealing their stance on Twitter (Figure S4).

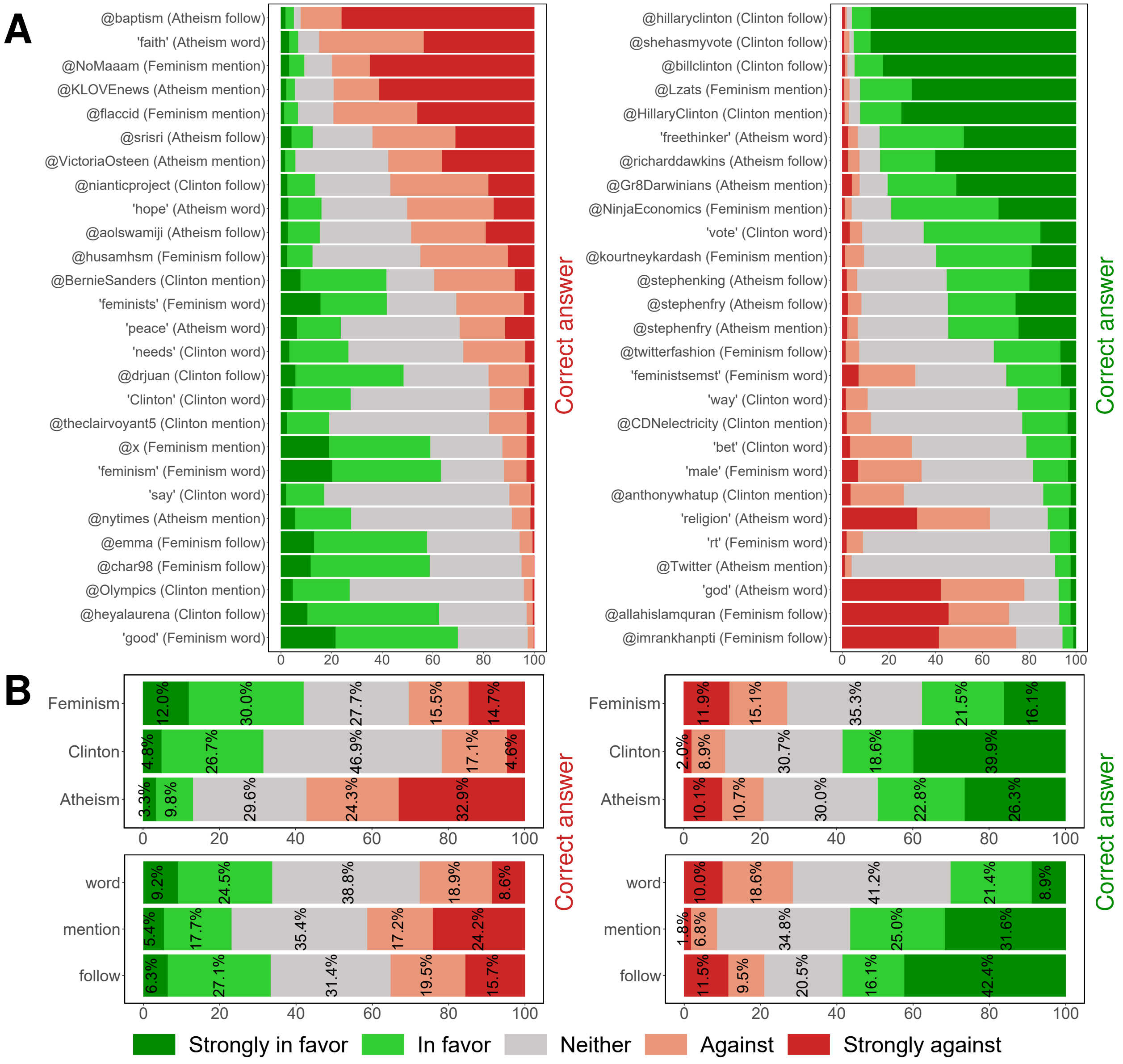

Figure S2: The same as Figure 2, but for each topic we exclude participants whose stance towards that topic is neutral.

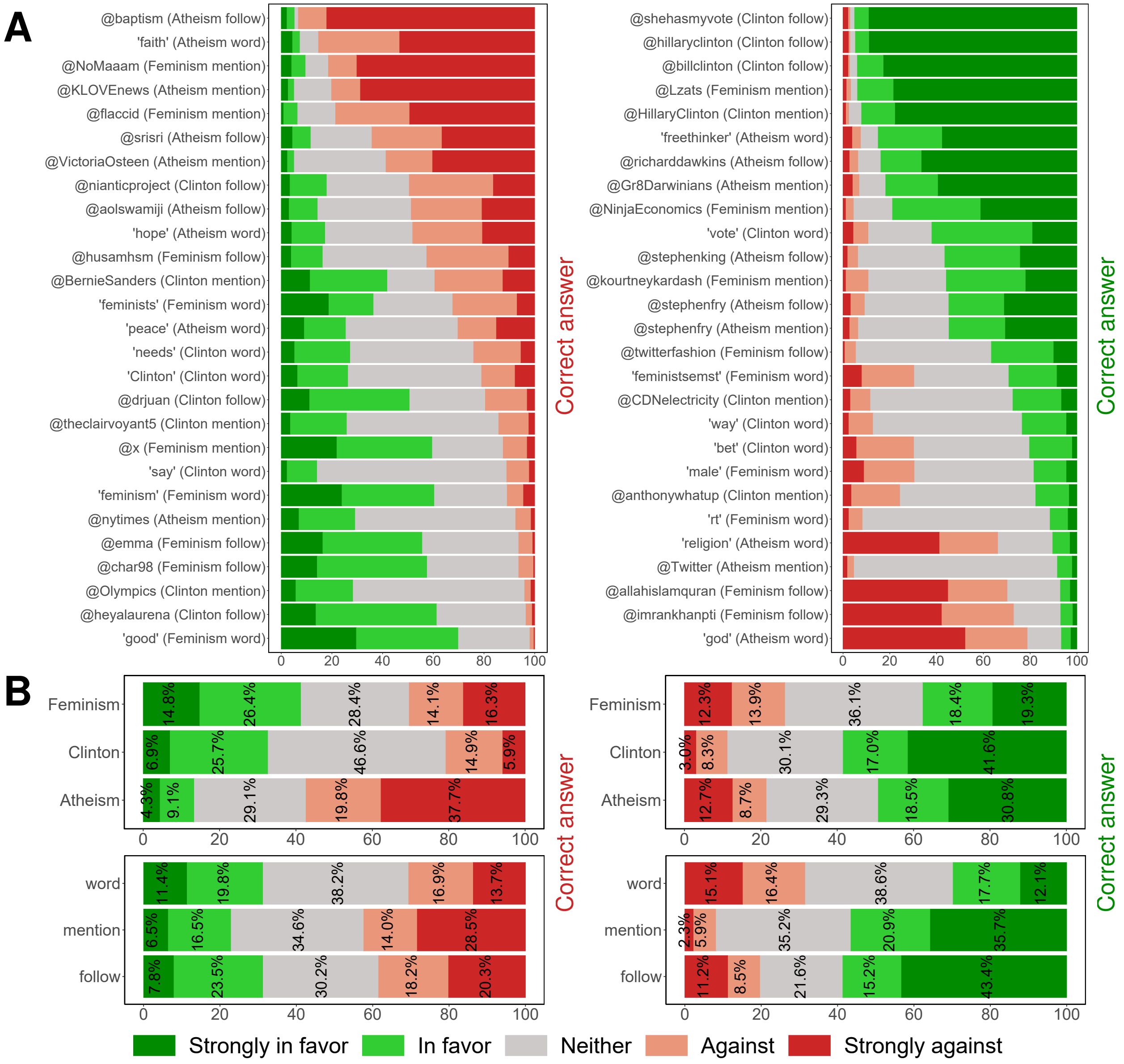

Figure S3: The same as Figure 2, but for each topic we consider only the participants whose are either strongly in favor or strongly against that topic.

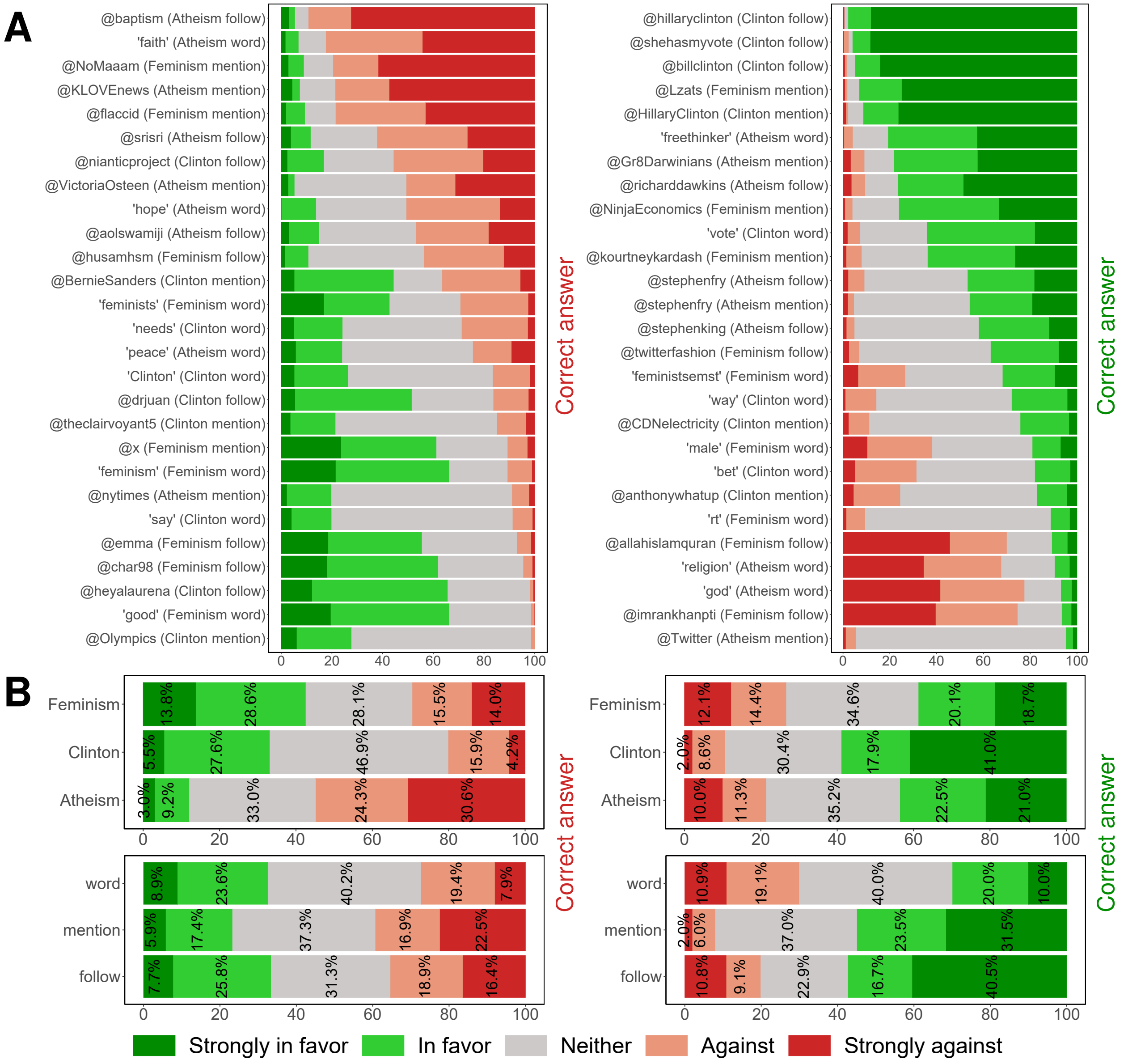

Figure S4: The same as Figure 2, but for each topic we consider only the participants who reported 8 or above when assessing their need to avoid revealing their stance towards that topic on Twitter.

S4 Computational Complexity of Optimal Hiding

In this section, we analyze the optimization problem faced by an automated assistant whose goal is to hide the evader's opinion from stance detection algorithms by modifying his/her Twitter account. We formulate this problem as follows:

Definition S1 (Optimal Stance Obfuscation)**.** *This problem is defined by a tuple, $(X, \mathbb{A}, c, b, f)$, where $X \in \mathbb{X}$ is a binary vector of the evader's features, $\mathbb{A}$ is a set of actions available to the evader, $c : \mathbb{A} \to \mathbb{R}$ is a function describing the cost of each action, $b \in \mathbb{R}$ is the budget of the evader specifying the total cost that the evader is willing to incur, and $f : \mathbb{X} \to \{0,1\}$ is a binary function that classifies the evader's stance based on their features. The goal is then to identify a set of actions $A^* \subseteq \mathbb{A}$ to be performed by the evader such that their total cost does not exceed b and the probability of the evader's stance being classified as 1 is minimized, i.e., A^* is in:*

$$\underset{A \subseteq \mathbb{A}: \sum_{a \in A} c(a) \leq b}{\arg\min} \quad P\left(f(X_A) = 1\right)$$

where X_A is the vector of the evader's features after performing the actions in A.

Here, the evader's goal is for their stance to be classified as 0 instead of 1 by the stance detection algorithm f. We introduce the notion of "cost" to reflect the fact that the evader may be more willing to perform certain actions than others. We also introduce the notion of "budget" to reflect the fact that the evader may have a limit on the modifications they are willing to make to their Twitter profile in return for their privacy.

Next, we analyze the computational complexity of the Optimal Stance Obfuscation problem. In our analysis, we focus on "k-nearest neighbors" as the stance detection algorithm from which the evader wishes to hide. We choose this algorithm not only due to its popularity as a general-purpose classifier, but also due to its closed-form formulation which makes it amenable to theoretical analysis. Despite the simplicity of this algorithm, the following theorem shows that the corresponding problem is NP-complete.

Theorem S1. *The Optimal Stance Obfuscation problem is NP-complete given the k-nearest neighbors algorithm when the set of actions is limited either to feature addition or to feature removal.*

Proof. Assuming that the actions of the evader can be performed in polynomial time and that the measure of distance used in the k-nearest neighbors algorithm can be computed in polynomial time, the problem is trivially in NP since the algorithm's outcome can be computed in polynomial time.

We will now show that the problem is NP-hard. To this end, we will show a reduction from the NP-complete 3-SAT problem. An instance (Y, C) of this problem is defined by a set of n variables $Y = \{y_1, \ldots, y_n\}$ and a conjunction of m clauses $C = C_1 \wedge \ldots \wedge C_m$, where each clause C_i is a disjunction of exactly 3 literals from $y_1, \ldots, y_n, \neg y_1, \ldots, \neg y_n$. The goal is to find an assignment of $\top$ and $\bot$ to variables in Y such that the conjunction C is satisfied.

We will first focus on the setting where the set of actions is limited to feature addition, after which we will describe the modifications of the proof necessary for the case of feature removal.

Based on the given instance of the 3-SAT problem, we will construct an instance of the Optimal Stance Obfuscation problem, and show a correspondence between the optimal solutions of both instances, thereby showing the NP-hardness. To this end, let (Y, C) be the given instance of the 3-SAT problem, where $Y = \{y_1, \ldots, y_n\}$ and $C = C_1 \wedge \ldots \wedge C_m$. We assume that no clause contains both negative and positive literal of the same variable. Based on (Y, C), we construct an instance $(X, \mathbb{A}, c, b, f)$ of the Optimal Stance Obfuscation problem as follows (Figure S5 presents an example of the construction and the reduction process):

- the initial vector of the evader's features is X such that $\forall_i X[i] = 0$ and $|X| = 2n$, i.e., it is a vector of $2n$ zeroes;
- the set of actions $\mathbb{A} = \{a_1, \ldots, a_n, \bar{a}_1, \ldots, \bar{a}_n\}$, where a_i is the action that involves setting the value of $X[i]$ to 1, while $\bar{a}_i$ is the action that involves setting the value of $X[n+i]$ to 1;
- the cost of every action is 1, i.e., $\forall_i c(a_i) = 1$;
- the budget of the evader is $b = n$;

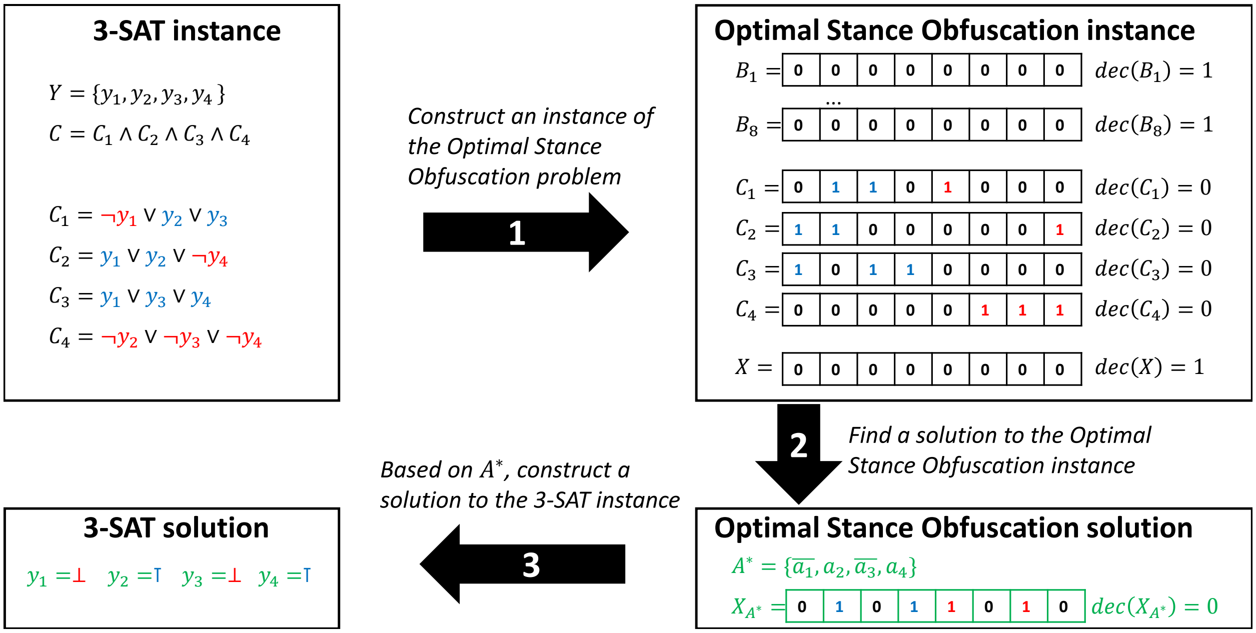

Figure S5: An example of the reduction from the 3-SAT problem to the Optimal Stance Obfuscation problem as described in the proof of Theorem S1, where the set of evader's actions is limited to feature addition. Solutions to both problems are highlighted in green, while the corresponding positive and negative elements of both problems are highlighted in blue and red, respectively.

- f is the k-nearest neighbors algorithm.

As for the details of the algorithm f, we assume that $k = 2m - 1$, and assume that the algorithm uses the following distance measure:

$$d(X, X') = 2n - \sum_{i=1}^{n} X[i]X'[i]\,(1 - X[n+i])\left(1 - X'[n+i]\right)$$
$$- \sum_{i=1}^{n} X[n+i]X'[n+i]\,(1 - X[i])\left(1 - X'[i]\right)$$

We also assume that the set of training examples consist of:

- $2m$ examples $B_1, \dots, B_{2m}$ where $\forall_i \forall_j B_i[j] = 0$ and $\forall_i |B_i| = 2n$, i.e., every B_i is a vector of $2n$ zeroes;
- m examples $C_1, \dots, C_m$ where for clause C_i and for $j \leq n$ we set:

$$C_i[j] = \begin{cases} 1 & \text{if clause } C_i \text{ contains literal } x_j \\ 0 & \text{otherwise} \end{cases}$$

and for $j > n$ we set $C_i[n+1] = C_i[n+2] = 1$,

$$C_i[n+j] = \begin{cases} 1 & \text{if clause } C_i \text{ contains literal } \neg x_j \\ 0 & \text{otherwise.} \end{cases}$$

The decision for every B_i is 1, while the decision for every C_i is 0.

Notice that given the set of actions $\mathbb{A}$, the budget b and the initial features vector X, the evader can set any n positions of the vector X to 1, while keeping the rest as 0. Intuitively, setting the i-th position to 1 for $i \leq n$

corresponds to choosing $y_i = \top$ in the given instance of the 3-SAT problem, while setting the $n + i$-th position to 1 corresponds to choosing $y_i = \bot$.

Notice also that for a given $A \subseteq \mathbb{A}$ the distance between X_A and any of the examples B_i is $d(X_A, B_i) = 2n$. At the same time, the distance between X_A and a given example C_i is $d(X_A, C_i) = 2n - \mu_i$, where μ_i is the number of positions j such that either:

- $j \leq n$, $C_i[j] = 1$ and $C_i[n + j] = 0$ (because of how C_i is constructed, this would be the case if and only if C_i contains the literal x_j), $X_A[j] = 1$ and $X_A[n + j] = 0$, or
- $j > n$, $C_i[j] = 1$ and $C_i[j - n] = 0$ (because of how C_i is constructed, this would be is the case if and only if C_i contains the literal $\neg x_{j-n}$), $X_A[j] = 1$ and $X_A[j - n] = 0$.

In such a situation we will say that X_A and C_i *match* in position j. Hence, the distance between X_A and a given C_i is smaller than the distance between X_A and any B_i if and only if X_A and C_i match in at least one position $j \leq n$. Since the algorithm f is the k-nearest neighbors with $k = 2m - 1$, and since there are $2m$ examples B_i (with decision 1) and only m examples C_i (with decision 0), the algorithm f assigns to X_A the decision 0 (desirable for the evader) with maximal probability if and only if X_A matches with all examples C_i in a least one position.

To prove the NP-hardness, we will now show that the constructed instance of the Optimal Stance Obfuscation problem has a solution if and only if the given instance of the 3-SAT problem has a solution.

Assume that there exists a solution y^* to the given instance of the 3-SAT problem, i.e., an assignment of values to variables y_i such that all clauses in C are satisfied, and let y_i^* denote the value assigned to y_i in this solution. Moreover, let A^* be the set of actions such that $a_i \in A^*$ if and only if $y_i^* = \top$ and $\bar{a}_i \in A^*$ if and only if $y_i^* = \bot$. Since the assignment y^* is a solution to the given instance of the 3-SAT problem, for every clause C_i there exists either a literal y_j in this clause such that $y_j^* = \top$, or a literal $\neg y_j$ in this clause such that $y_j^* = \bot$. In the first case we have $C_i[j] = 1$, $C_i[n + j] = 0$, $X_{A^*}[j] = 1$ and $X_{A^*}[n + j] = 0$, while in the second case we have $C_i[n + j] = 1$, $C_i[j] = 0$, $X_{A^*}[n + j] = 1$ and $X_{A^*}[j] = 0$. Therefore, X_{A^*} matches with every example C_i in at least one position, and it is assigned the decision 0 by the algorithm f with probability 1. Hence, A^* is a solution to the constructed instance of the Optimal Stance Obfuscation problem.

To prove the other implication, assume that there exists a solution A^* to the constructed instance of the Optimal Stance Obfuscation problem. Let $y_i^* = \top$ if $a_i \in A^* \wedge \bar{a}_i \notin A^*$, and let $y_i^* = \bot$ if $\bar{a}_i \in A^* \wedge a_i \notin A^*$. Otherwise, assign the value of y_i^* randomly (as it is not crucial for satisfying the constraints in the 3-SAT problem instance). Since the algorithm f assigns the decision 0 to X_{A^*} with probability 1, it must match with every C_i in at least one position, i.e., for every C_i there exists j such that either:

- $j \leq n$, $C_i[j] = 1$, $C_i[n + j] = 0$, $X_A[j] = 1$ and $X_A[n + j] = 0$, or
- $j > n$, $C_i[j] = 1$, $C_i[j - n] = 0$, $X_A[j] = 1$ and $X_A[j - n] = 0$.

Because of how C_i and y_j^* are constructed, in the first case we have that clause C_i in the given instance of the 3-SAT problem contains literal y_j and $y_j^* = \top$, while in the second case we have that clause C_i contains literal $\neg y_j$ and $y_j^* = \bot$. Hence, the assignment y_i^* satisfies every clause C_i in C, which makes it a solution to the given instance of the 3-SAT problem.

Therefore, we showed that the constructed instance of the Optimal Stance Obfuscation problem has a solution if and only if the given instance of the 3-SAT problem has a solution. This concludes the proof for the case where the set of actions is limited to feature addition.

In order to obtain the proof for the case of the feature removal, we have to perform the following modifications:

- the initial vector of the evader's features is X such that $\forall_i X[i] = 1$ and $|X| = 2n$, i.e., it is a vector of $2n$ ones;
- the set of actions $\mathbb{A} = \{a_1, \ldots, a_n, \bar{a}_1, \ldots, \bar{a}_n\}$, where a_i corresponds to setting the value of $X[n + i]$ to 0, while $\bar{a}_i$ corresponds to setting the value of $X[i]$ to 0.

The remainder of the proof follows exactly the same reasoning. □

S5 Evaluating Cross-topic Implications

In the main manuscript, we evaluated the impact of our heuristic on a given topic, without considering the possible side-effect of accidentally influencing one's perceived stance towards the other two topics. In this section, we run a set of experiments intended to analyze such cross-topic implications. To this end, we focus on different subsets of topics; these subsets are: $\{\text{Atheism}, \text{Clinton}\}$, $\{\text{Atheism}, \text{Feminism}\}$, $\{\text{Clinton}, \text{Feminism}\}$, and $\{\text{Atheism}, \text{Feminism}, \text{Clinton}\}$. For each subset, we calculate the overlap between the features that indicate one's stance towards all the topics in that subset. Intuitively, if this overlap is relatively small, it suggests that changing the features that are related to a topic has a limited impact on the other topic(s).

In our cross-topic experiments, we took the 1000 most indicative features for each topic and each stance (in favor vs. against), and computed the overlap between these features for each subset of topics. The results of this analysis can be found in Figure S6. As expected, the overlap increases as we increase the number features taken into consideration. Consequently, to minimize the cross-topic influence, one must limit the number of features that are modified by the heuristic. For example, if the user only modifies 25 features—a modification that effectively hides their stance, as shown in Figure 3 in the main article—none of these 25 would appear among the top 25 features for the other topics. The only exception is the subset $\{\text{Clinton}, \text{Feminism}\}$, since the top 25 features related to Hilary Clinton and the top 25 features related to feminism have a single feature in common. These results suggest that there is a trade-off between hiding one's opinion towards a topic and affecting one's perceived opinion towards other topics. They also suggest that, as long as the hiding efforts are not excessive, then the cross-topic influence is likely to be limited.

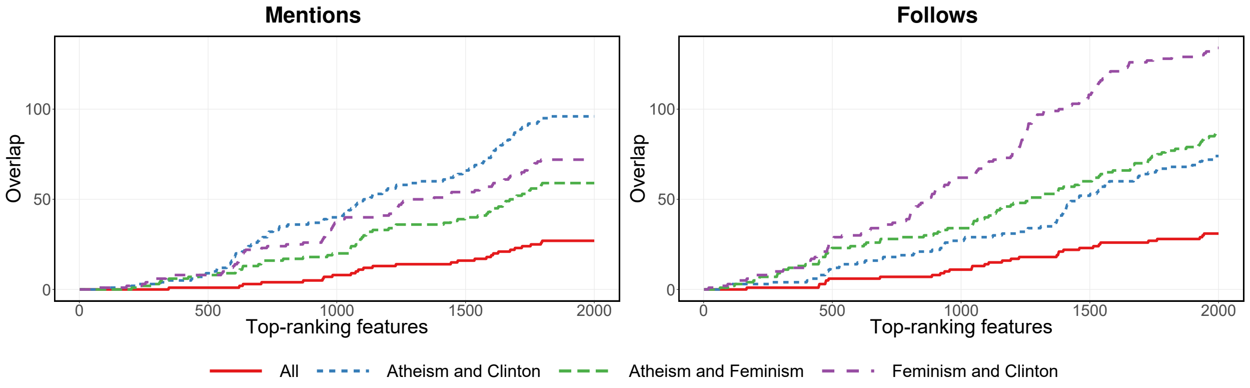

Figure S6: The overlap between the features that are most indicative of one's stance towards each topic. The x-axis represents the number of highest-ranked features that are considered in the analysis, the y-axis represents the overlap between those features, while different colors represent different subsets of topics. For example, the green line depicts the overlap between the features that indicate one's stance towards atheism and towards feminism. For each these two topics, $x = 100$ represents the 50 highest-ranked features indicating in-favor, and 50 highest-ranked features indicating against, resulting in a total of 100 features per topic. The y-axis would then represent the overlap between the 100 features related to atheism and the 100 features related to feminism.
